# Evaluating the reliability and educational quality of YouTube™ and TikTok™ videos on custom subperiosteal implants: a cross-sectional methodological analysis

**DOI:** 10.1186/s12903-026-08290-x

**Published:** 2026-04-07

**Authors:** Göksel Tımarcıoğlu, Erdal Cem Kargu, Hülya Çerçi Akçay, Başak Tımarcıoğlu

**Affiliations:** 1 Department of Oral and Maxillofacial Surgery, Faculty of Dentistry, Kocaeli Health and Technology University, Kocaeli, Turkey; 2 Faculty of Dentistry, Kocaeli Health and Technology University, Kocaeli, Turkey; 3 Department of Pedodontics, Kocaeli Health and Technology University, Kocaeli, Turkey; 4 Private Practice, Atasehir, Istanbul, Turkey

**Keywords:** YouTube, TikTok, Subperiosteal implant, Dental implantology, Video analysis, Global Quality Scale, DISCERN, JAMA benchmark, VIQI, Patient education

## Abstract

**Background:**

Social media platforms such as YouTube™ and TikTok™ have become increasingly influential sources of oral health information. However, the reliability and educational value of videos on advanced surgical procedures, including custom subperiosteal implants, remain uncertain. This study aimed to evaluate and compare the quality, accuracy, and educational usefulness of YouTube™ and TikTok™ videos related to custom subperiosteal implant procedures.

**Methods:**

This cross-sectional methodological study analyzed 38 videos (YouTube = 23, TikTok = 15) identified using English and Turkish keywords. Eligible videos were assessed independently by two calibrated reviewers using the Global Quality Scale (GQS), DISCERN, Journal of the American Medical Association (JAMA) benchmark criteria, and the Video Information and Quality Index (VIQI). A seven-domain content rubric specific to subperiosteal implant education was also applied. Descriptive statistics, Spearman correlation, and Kruskal–Wallis tests were conducted. Interrater agreement was evaluated using Weighted Kappa for GQS and DISCERN, and intraclass correlation coefficients (ICC) for VIQI, JAMA, and total scores.

**Results:**

Overall video quality was moderate, with mean scores of GQS = 2.66 ± 1.43, DISCERN = 32.71 ± 21.66, JAMA = 1.70 ± 0.54, and VIQI = 10.83 ± 4.90. Strong positive correlations were observed among GQS and DISCERN (*r* = 0.933, *p* < 0.001), GQS and VIQI (*r* = 0.942, *p* < 0.001), and DISCERN and VIQI (*r* = 0.949, *p* < 0.001), indicating substantial concordance among validated quality instruments. In contrast, total views demonstrated a strong negative correlation with JAMA scores (*r* = -0.748, *p* < 0.001), and likes were negatively correlated with JAMA (*r* = -0.822, *p* < 0.001), suggesting that higher viewer engagement was not associated with greater transparency or reliability. Interrater agreement was excellent across all tools (ICC > 0.79). No statistically significant differences were observed among uploader categories (*p* > 0.05), although videos uploaded by academic institutions and dentists demonstrated descriptively higher quality scores.

**Conclusions:**

YouTube™ and TikTok™ videos related to custom subperiosteal implants demonstrate moderate reliability and educational value, with substantial variability in content completeness. Video popularity did not reflect scientific accuracy. These findings should be interpreted in light of the limited sample size, the non-validated nature of the topic-specific rubric, and inherent differences between social media platforms. High-quality, evidence-based audiovisual resources created by dentists and academic institutions are needed to support patient education and informed decision-making.

**Trial registration:**

Not applicable.

## Background

Social media platforms have become widely used sources of health-related information for the general population, accompanied by substantial increases in user engagement and information-seeking behavior in recent years [1–3]. In this context, the internet has emerged as a major source of health information, and video-sharing platforms such as YouTube™ and TikTok™ are increasingly used to access dental content [4]. Their audiovisual nature, accessibility, and algorithm-driven exposure allow dental information to reach large audiences rapidly [5]. However, the unregulated structure of these platforms raises concerns regarding the reliability and educational accuracy of the information presented [6]. Unverified or misleading content may negatively influence patient decision-making, leading to misconceptions about dental procedures and compromising evidence-based care [7].

Within dentistry, social media plays an expanding role in patient education and professional communication [8]. Visually complex procedures—such as implantology, surgical interventions, and digital prosthetic planning—are particularly popular topics on these platforms [9]. Custom subperiosteal implants, digitally designed and manufactured using CAD/CAM workflows, have recently gained prominence due to their innovative and reconstructive nature [10]. As these implants are primarily indicated for patients with advanced alveolar bone resorption, accurate patient understanding of indications, benefits, limitations, and potential risks is essential [11].

Previous analyses of online dental videos have shown that although many attract high view counts, their scientific accuracy and objectivity vary considerably. Some videos emphasize aesthetic or promotional aspects rather than clinical accuracy. Nevertheless, evidence suggests that well-designed audiovisual materials can improve comprehension, retention, and adherence to clinical advice. Studies also indicate that visual learning enhances patient engagement more effectively than text-based information, highlighting the potential value of high-quality educational videos when developed responsibly [12, 13].

Despite the increasing availability of dental content online, few studies have evaluated TikTok™, a platform characterized by shorter but highly engaging video formats, particularly in comparison with YouTube™. Given the rapid growth of these platforms and the limited evidence regarding advanced surgical procedures such as custom subperiosteal implants, there is a need to investigate the reliability and educational accuracy of the content available to the public.

Therefore, this study aimed to evaluate and compare the reliability, quality, and educational value of YouTube™ and TikTok™ videos related to custom subperiosteal implant procedures using validated assessment tools.

## Methods

### Study design and objective

This descriptive, cross-sectional study aimed to evaluate the reliability, accuracy, and educational quality of YouTube™ and TikTok™ videos related to custom subperiosteal implants. The methodological framework was adapted from previously validated video-based analyses in dental research to ensure consistency and reproducibility [14, 15]. The primary objective was to determine whether audiovisual materials available on these platforms provide scientifically accurate and valuable information for patients and the general public seeking health information. Accordingly, the evaluation and interpretation of results were conducted within a patient-centered framework, consistent with the intended scope of consumer-oriented assessment tools such as DISCERN and the Global Quality Scale. However, the overall evaluation framework was multidimensional, incorporating transparency (JAMA benchmark criteria) and audiovisual/technical quality (VIQI) dimensions in addition to patient-centered informational quality. This integrated approach allowed a more comprehensive assessment of scientific reliability and educational value.

### Search strategy

A systematic search was performed on YouTube™ (www.youtube.com) and TikTok™ (www.tiktok.com) on October 28, 2025. All searches were conducted using a clean browser without login, cookies, or prior search history to minimize algorithmic bias. For YouTube™, the relevance filter was selected, while TikTok™ searches were conducted using the default “overall ranking” mode, which reflects the content most commonly displayed to users.

Five English and Turkish keywords were used to capture both lay and professional terminology: “subperiostal implant,” “subperiostal implant uygulaması,” “kişiye özel subperiostal implant,” “custom subperiosteal implant,” and “subperiosteal dental implant.”

The comprehensive search initially yielded approximately 2,326 videos across both platforms. However, consistent with established methodology in social media video analyses, only the first 140 algorithmically ranked videos were screened. These results represent the content most likely to be encountered by typical users under default relevance-based ranking. This predefined screening limit was applied to ensure methodological consistency, real-world relevance, and reproducibility while minimizing algorithmic drift across search sessions.

**Fig. 1:**
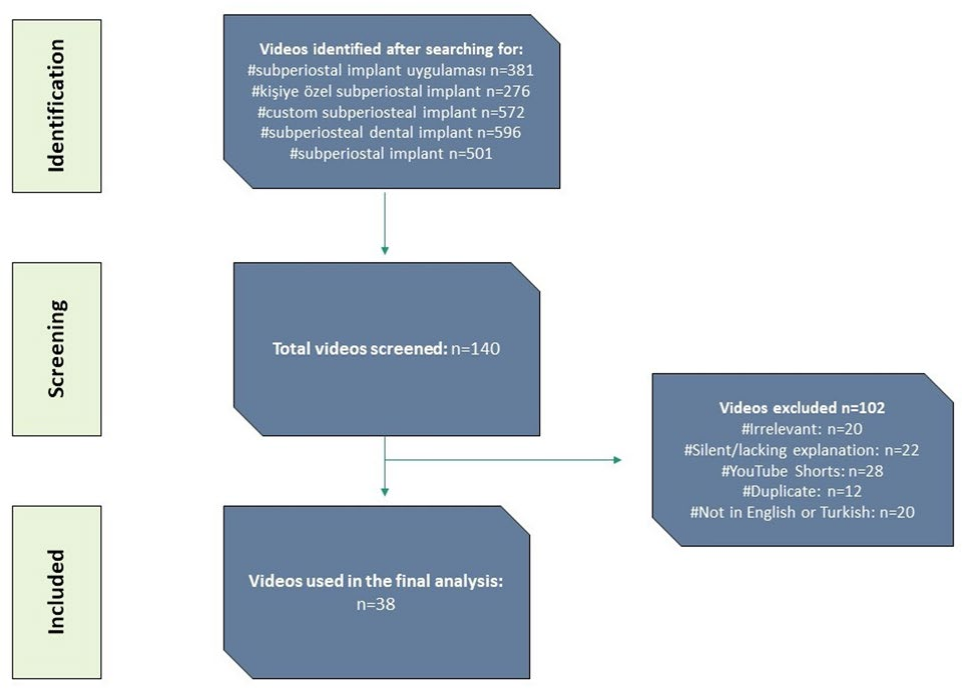
Flowchart illustrating the selection process of YouTube™ and TikTok™ videos. The initial search yielded approximately 2,326 videos; however, only the first 140 algorithmically ranked results were screened in accordance with predefined methodological limits

After removal of duplicates and application of predefined eligibility criteria, 38 videos were included in the final analysis (YouTube™ = 23; TikTok™ = 15). The full selection process is illustrated in Fig. 1 in accordance with PRISMA-style reporting.

All searches were conducted from Turkey using a local IP address; therefore, the retrieved results may reflect region-specific algorithmic behavior, particularly on TikTok™.

### Eligibility criteria

Videos were included if they:

- were in English or Turkish,
- contained narration or captions,
- provided information related to the design, digital planning, surgical steps, or prosthetic restoration of custom subperiosteal implants, and.
- were uploaded by dentists, academic institutions, health websites or other sources.

Videos were excluded if they:

- were unrelated to subperiosteal implantology,
- lacked verbal or textual explanation,
- were shorter than 15 s (e.g., YouTube™ Shorts, TikTok™ Reels),
- were duplicates, or.
- were presented in languages other than English or Turkish.

A total of 102 videos were excluded based on these criteria (Fig. 1).

### Data collection and coding

For each included video, descriptive information such as title, upload date, duration, number of views, likes, comments, and uploader categories was recorded in a structured Excel spreadsheet. Uploaders were classified as individual dentists, academic institutions, health websites, or other sources.

Viewer engagement was assessed using only publicly available and directly verifiable metrics. Accordingly, engagement was quantified using the like ratio (likes/views) and engagement rate ((likes + comments)/views). Metrics relying on dislike counts were not used, as YouTube™ no longer provides public access to dislike data.

For TikTok™ videos, certain engagement metrics (e.g., dislikes and shares) are not publicly available. Therefore, direct quantitative comparisons of engagement metrics between YouTube™ and TikTok™ were not intended. TikTok™ content was evaluated descriptively using validated educational quality assessment tools only (GQS, DISCERN, JAMA, and VIQI).

Video duration data were fully available for YouTube™ videos but inconsistently reported or not publicly verifiable for TikTok™ content. An exploratory, platform-restricted correlation analysis between video duration and quality scores was initially considered for YouTube™ videos; however, given the limited sample size and the descriptive, cross-platform focus of the study, these exploratory analyses were not included in the final statistical evaluation to avoid overinterpretation.

All data were publicly available and independently verified by two reviewers (GT and ECK) to ensure accuracy and consistency.

### Video content and quality assessment

Each video was evaluated using a seven-domain, topic-specific rubric designed to assess the educational completeness of subperiosteal implant–related content. The rubric encompassed the following domains: definition, indications and contraindications, procedure stages, advantages and disadvantages, comparison with other techniques, follow-up, and patient information.

These domains were selected to reflect clinically relevant educational components of subperiosteal implant procedures and to address commonly reported information gaps in previous video-based dental education studies [14, 15]. Each domain was rated using a five-point Likert scale (1 = very poor, 5 = excellent), yielding a total possible score ranging from 7 to 35. Formal validation and internal consistency testing of the rubric were not performed and are acknowledged as a limitation of the present study.

The topic-specific rubric was used to provide supplementary descriptive insight into content completeness and was not intended to support inferential conclusions. All primary comparisons and interpretations were based on validated instruments (GQS, DISCERN, JAMA, and VIQI).

Four validated instruments were also used to evaluate reliability and overall educational quality:

- Global Quality Scale (GQS),
- DISCERN instrument,
- Journal of the American Medical Association (JAMA) benchmark criteria,
- Video Information and Quality Index (VIQI).

All evaluations were conducted independently by two calibrated reviewers, and discrepancies were resolved through consensus.

### Reviewer calibration and reliability

Before beginning the full assessment, both reviewers completed a calibration phase involving 10 randomly selected videos (five from each platform). Interrater agreement for ordinal scales (GQS and DISCERN) was assessed using weighted Cohen’s kappa, whereas continuous scores (JAMA, VIQI, and total score) were evaluated using ICC (two-way mixed-effects model, absolute agreement, single measures) [16–19]. Agreement was interpreted as poor (< 0.50), moderate (0.50–0.75), good (0.75–0.90), or excellent (> 0.90).

### Statistical analysis

Descriptive statistics, normality testing (Shapiro–Wilk), correlation analyses (Spearman), and group comparisons (Kruskal–Wallis) were performed using IBM SPSS Statistics v29.0. Hierarchical cluster analysis (Ward’s method) and graphical visualizations were conducted in Python v3.11 using SciPy and Seaborn libraries. A post-hoc power analysis based on observed effect sizes indicated adequate statistical power (> 80%) to detect moderate correlations. Post-hoc power analysis was included for descriptive purposes only and should be interpreted cautiously, as effect sizes and confidence intervals provide more informative measures of observed associations.

Normality was assessed using the Shapiro–Wilk test. Due to non-normal distribution of data, non-parametric statistical tests were applied.

Descriptive statistics were reported as mean ± standard deviation (SD) with 95% confidence intervals (CI) and median with interquartile range (Q3–Q1).

Interrater reliability was assessed using Weighted Kappa for GQS and DISCERN, and ICC (two-way mixed-effects, absolute agreement) for JAMA, VIQI, and total scores.

Correlations among quality indices and engagement metrics (views, likes) were analyzed using Spearman’s rank correlation. Differences among uploader categories were assessed using the Kruskal–Wallis test. Hierarchical cluster analysis (Ward’s method) was performed to identify natural groupings of videos based on quality profiles.

A p-value < 0.05 was considered statistically significant.

## Results

A total of 140 videos were identified during the initial search. After removal of duplicates and application of inclusion and exclusion criteria, 38 videos were included in the final analysis (YouTube™ = 23; TikTok™ = 15). The selection process is presented in Fig. 1.

### Video characteristics and descriptive findings

Descriptive statistics of video features and content-related rubric scores are presented in Table 1. Total view counts and like numbers demonstrated wide variability across both platforms, indicating heterogeneous audience exposure and engagement.

**Table 1:** Descriptive statistics regarding the general features and content quality scores of subperiosteal implant videos

Variable	Mean ± SD (95% CI)	Median (Q1–Q3)
Total Number of Views	2356.65 ± 2730.59 (1397.80–3450.32)	1523.00 (665.50-2836.50)
Total Number of Likes	33.56 ± 39.37 (19.61–52.27)	19.00 (9.00–45.00)
Definition	2.39 ± 1.58 (1.95–2.87)	1.50 (1.00–4.00)
Indications/Contraindications	2.08 ± 1.37 (1.68–2.53)	1.00 (1.00-3.38)
Procedure stages	2.86 ± 1.51 (2.39–3.32)	2.75 (1.25-4.00)
Advantages/Disadvantages	2.20 ± 1.39 (1.80–2.63)	2.00 (1.00–3.00)
Comparison with other techniques	2.05 ± 1.36 (1.67–2.50)	1.00 (1.00–3.00)
Follow-up	1.72 ± 0.93 (1.45–2.01)	1.00 (1.00–2.00)
Patient information	2.47 ± 1.40 (2.03–2.96)	2.00 (1.00–4.00)
TOTAL	16.39 ± 9.15 (13.39–19.16)	12.00 (8.25-26.00)

Data are presented as mean ± standard deviation (SD), 95% confidence interval (CI), and median with interquartile range (Q1-Q3). Only publicly available engagement metrics (views and likes) were included. Descriptive analyses were performed using non-parametric methods due to non-normal data distribution

Mean values for the educational and quality assessment tools were as follows:

- Global Quality Scale (GQS): 2.66 ± 1.43
- DISCERN: 32.71 ± 21.66
- JAMA benchmark score: 1.70 ± 0.54
- Video Information and Quality Index (VIQI): 10.83 ± 4.90

Overall, these results indicate moderate educational and structural quality, with considerable variation among videos. Detailed descriptive statistics for each instrument are provided in Table 2.

**Table 2:** Descriptive statistics for video characteristics, content quality domains, and evaluation scores (GQS, DISCERN, JAMA Benchmark, VIQI)

	Mean ± SD (95% CI)	Median (Q1–Q3)
Global Quality Scale (GQS)	2.66 ± 1.43 (2.24–3.07)	3.00 (1.00-3.88)
DISCERN	32.71 ± 21.66 (25.51–39.50)	33.00 (12.00-53.62)
JAMA Benchmark	1.70 ± 0.54 (1.53–1.88)	2.00 (1.00–2.00)
Video Information and Quality Index (VIQI)	10.83 ± 4.90 (9.29–12.34)	11.00 (7.00-15.38)

Values are presented as mean ± SD (95% CI) and median (Q1–Q3)

### Interrater reliability

Inter-rater agreement between the two reviewers was excellent across all evaluation tools. Weighted Kappa values for GQS and DISCERN exceeded 0.90, while ICC values for JAMA, VIQI, and total scores ranged from 0.79 to 0.99 (all *p* < 0.001). These findings confirm the consistency and reproducibility of the evaluation process (Table 3).

**Table 3:** Interrater agreement for video evaluation scales assessed using weighted Cohen’s kappa and intraclass correlation coefficient (ICC)

Scale	ICC/Weighted Kappa	95% CI	*P* Value
Global Quality Scale (Weighted Kappa)	0.901	(0.803–0.980)	< 0.001
DISCERN Score (Weighted Kappa)	0.922	(0.860–0.943)	< 0.001
JAMA (ICC)	0.793	(0.638–0.886)	< 0.001
Video Information and Quality Index (ICC)	0.948	(0.903–0.973)	< 0.001
Total Score (ICC)	0.994	(0.989–0.997)	< 0.001

Weighted Cohen’s kappa was used to assess interrater agreement for ordinal scales (Global Quality Scale and DISCERN), whereas intraclass correlation coefficient (ICC; two-way mixed-effects model, absolute agreement, single measures) was applied for continuous scores (JAMA, VIQI, and total score).

### Correlation analysis

Correlation analyses were performed using only raw, publicly verifiable engagement metrics, specifically total views and total likes. Derived metrics such as like ratio or engagement rate were calculated for descriptive purposes but were not included in correlation testing.

Spearman correlation analysis revealed strong positive correlations between GQS and DISCERN (*r* = 0.933, *p* < 0.001), GQS and VIQI (*r* = 0.942, *p* < 0.001), and DISCERN and VIQI (*r* = 0.949, *p* < 0.001). DISCERN also demonstrated a near-perfect correlation with total score (*r* = 0.992, *p* < 0.001).

In contrast, total views showed a strong negative correlation with JAMA scores (*r* = -0.748, *p* < 0.001), and likes were negatively correlated with JAMA (*r* = -0.822, *p* < 0.001).

**Fig. 2:**
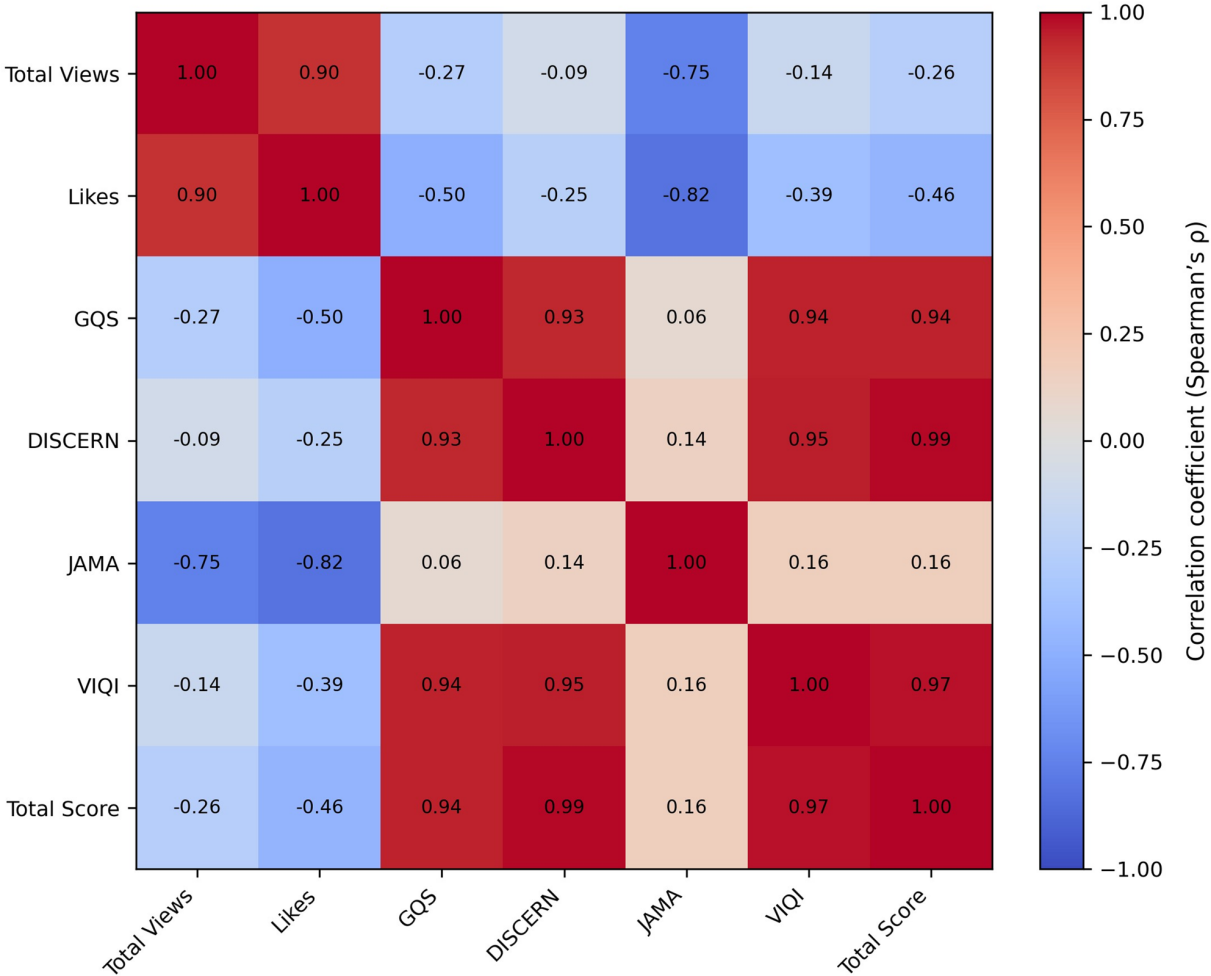
Spearman correlation heatmap illustrating the relationships between engagement metrics (total views, likes) and content quality scores (GQS, DISCERN, JAMA, VIQI, and total score)

These findings indicate that higher viewer engagement was not associated with higher scientific transparency or reliability.Correlation findings are visualized Fig. 2 and detailed in Table 4.

**Table 4:** Correlation analysis among scientific quality scales

	Total Views	Likes	GQS	DISCERN	JAMA	VIQI	Total Score
Total Views	—	0.897**	-0.267	-0.088	-0.748**	-0.137	-0.264
Likes	0.897**	—	-0.495	-0.249	-0.822**	-0.391	-0.459
GQS	-0.267	-0.495	—	0.933**	0.061	0.942**	0.944**
DISCERN	-0.088	-0.249	0.933**	—	0.137	0.949**	0.992**
JAMA	-0.748**	-0.822**	0.061	0.137	—	0.164	0.163
VIQI	-0.137	-0.391	0.942**	0.949**	0.164	—	0.972**
Total Score	-0.264	-0.459	0.944**	0.992**	0.163	0.972**	—

Spearman correlation test was used. Only publicly available engagement metrics (total views and likes) were included in the analysis. *p < 0.05, **p < 0.01 were considered statistically significant. Exact *p*-values for significant correlations were all < 0.001

### Platform-based comparison

When videos were compared according to platform (YouTube™ vs. TikTok™), YouTube™ videos demonstrated significantly higher GQS (U = 328.0, *p* < 0.001), DISCERN (U = 337.5, *p* < 0.001), and VIQI scores (U = 335.5, *p* < 0.001) compared with TikTok™ videos. No statistically significant difference was observed in JAMA benchmark scores between platforms (U = 170.0, *p* = 0.948).

### Comparison by uploader categories

**Fig. 3:**
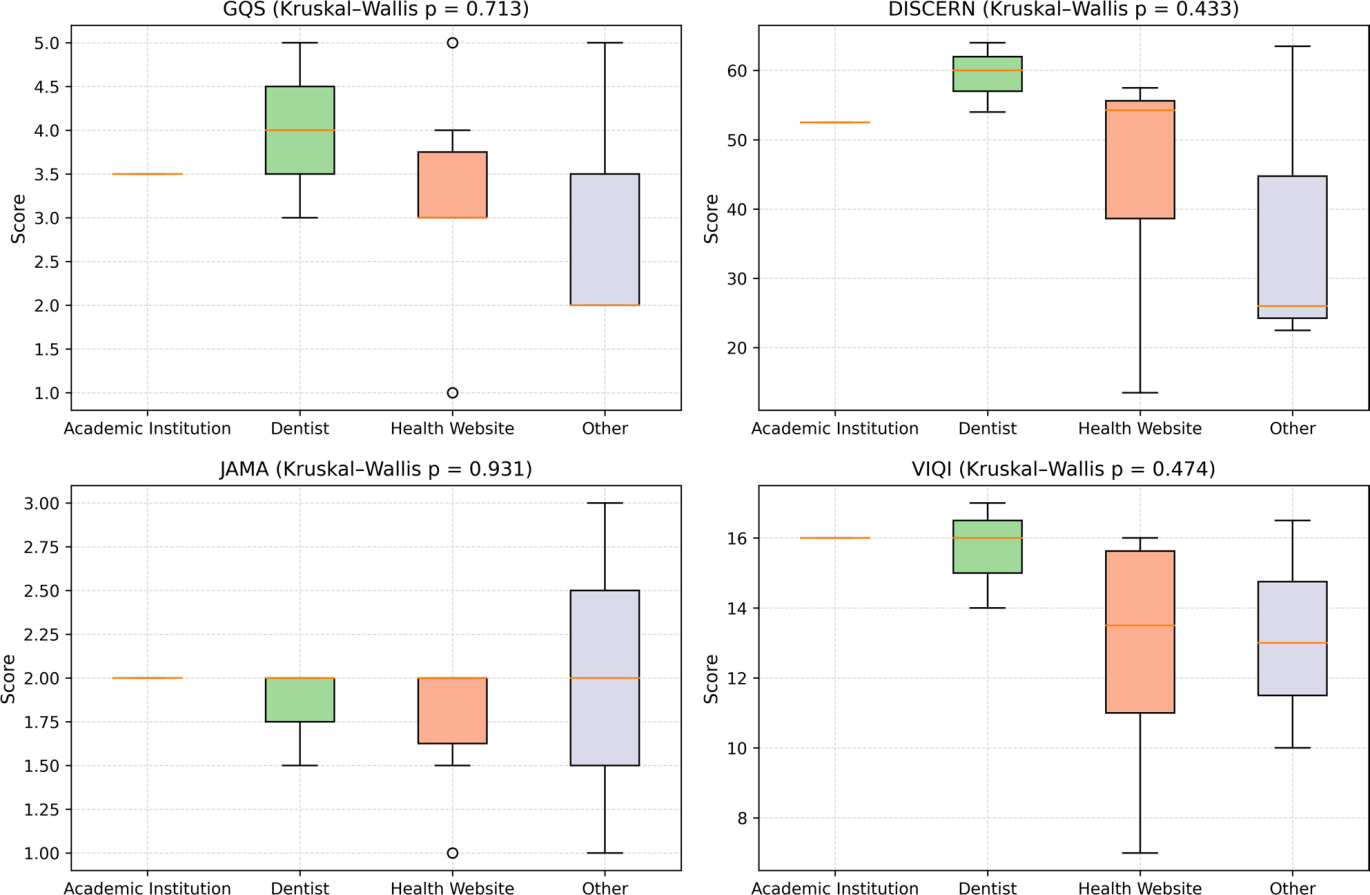
Comparison of content quality scores (GQS, DISCERN, JAMA, and VIQI) across uploader categories based on the Kruskal–Wallis test

The Kruskal–Wallis analysis revealed no statistically significant differences in GQS, DISCERN, JAMA, or VIQI scores across uploader categories (*p* > 0.05). However, descriptive trends showed that videos uploaded by academic institutions and dentists tended to have slightly higher median quality scores. These comparisons are illustrated in Fig. 3.

### Cluster analysis

**Fig. 4:**
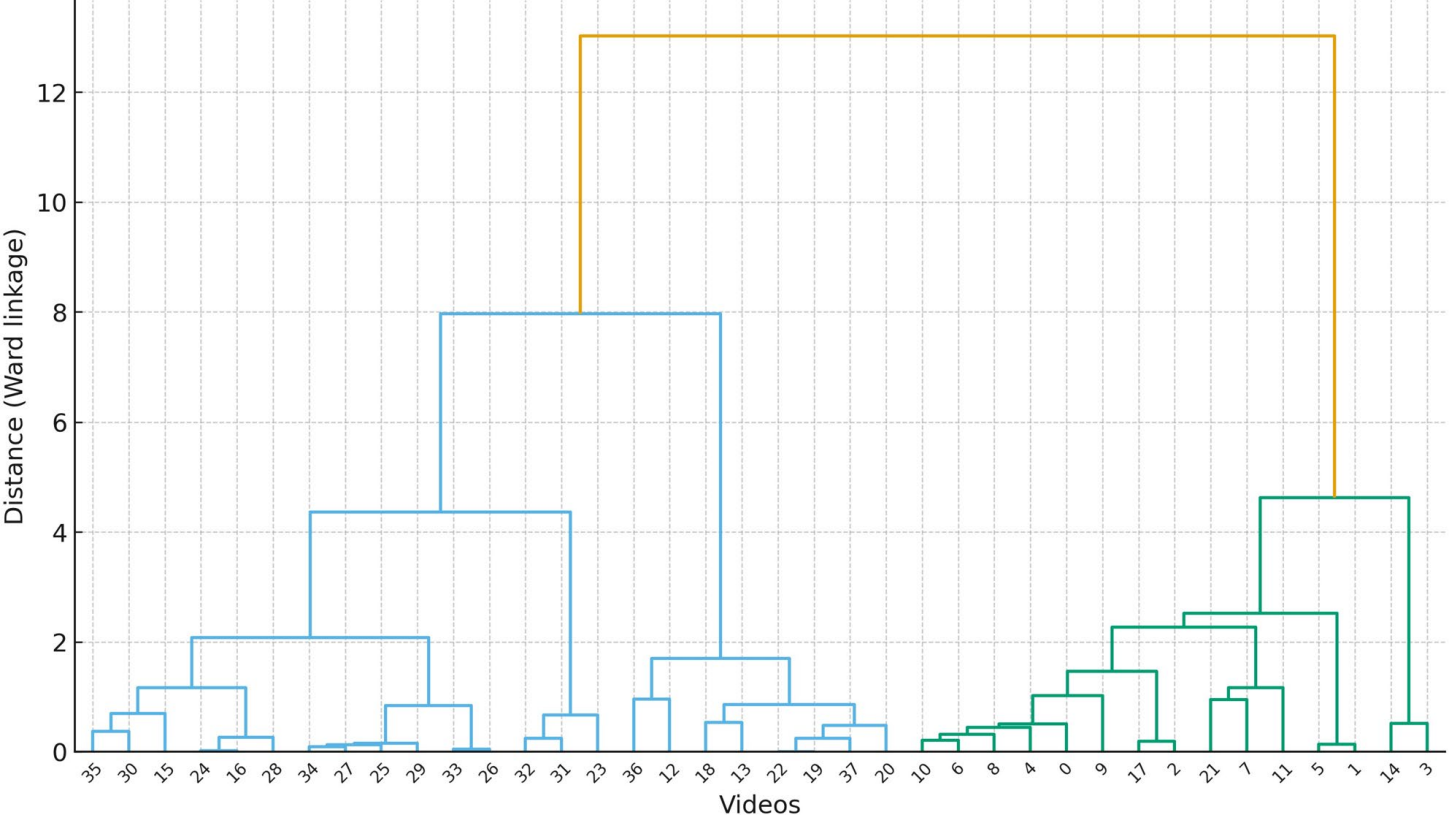
Hierarchical clustering dendrogram (Ward’s method) showing exploratory grouping of videos based on GQS, DISCERN, JAMA, and VIQI scores. Two main clusters were identified, broadly separating higher- and lower-quality content

Hierarchical cluster analysis (Ward’s method) identified two primary video clusters based on overall quality profiles. One cluster contained videos with consistently higher GQS, DISCERN, JAMA, and VIQI scores, primarily uploaded by academic institutions or dentists, whereas the other cluster included lower-quality videos from non-professional or commercial accounts. The clustering structure is shown in Fig. 4.

## Discussion

This study evaluated and compared the reliability, quality, and educational value of YouTube™ and TikTok™ videos related to subperiosteal implant procedures. Overall, although both platforms host a substantial volume of content on this topic, the educational quality and informational reliability of the available videos were highly variable. YouTube™ videos demonstrated significantly higher DISCERN, GQS, and VIQI scores than TikTok™ videos, whereas no statistically significant difference was observed in JAMA benchmark scores.This divergence reflects a frequently reported discrepancy between popularity and educational depth in online dental content and supports interpretation of the findings within a patient-centered information framework rather than a professional training perspective [20].

A particularly important finding of this study is the observed negative association between video popularity metrics, especially total views, and scientific quality scores. This pattern reflects a “popularity–quality paradox,” whereby the most widely viewed content is not necessarily the most reliable or educational. From a public health perspective, this phenomenon is concerning, as platform algorithms may preferentially amplify visually appealing or simplified content at the expense of accuracy, transparency, and balanced discussion of risks and alternatives. Consequently, patients seeking information on complex procedures such as custom subperiosteal implants may be disproportionately exposed to lower-quality content, potentially shaping unrealistic expectations and incomplete understanding. These findings highlight the critical need for academic institutions and professional organizations to actively participate in the creation and algorithm-aware dissemination of evidence-based audiovisual educational materials.

Consistent with prior research on orthodontic, restorative, and bleaching-related content, social media videos frequently fail to meet established educational standards, often prioritizing promotional or procedural visuals over comprehensive scientific explanation [21–24]. Although professionally produced videos generally achieved higher quality scores, dentist-based or academic authorship alone did not guarantee transparency, completeness, or sufficient informational depth. This imbalance between engagement-driven visibility and content reliability continues to undermine the effectiveness of social media as a tool for patient education.

In the specific context of subperiosteal implants, these limitations carry important clinical implications. Subperiosteal implant therapy is typically indicated in patients with advanced alveolar bone resorption or conditions unsuitable for endosseous implants and therefore requires thorough understanding of digital planning, surgical execution, anatomical considerations, and postoperative care [25, 26]. However, many of the analyzed videos focused predominantly on visual aspects of the procedure while omitting essential educational elements such as patient selection criteria, contraindications, complication risks, and long-term follow-up [27]. Scores derived from Likert-based instruments, particularly GQS and VIQI, reflected these deficiencies, with frequent low performance in domains related to definition clarity, information flow, and patient education. Notably, omission of safety-related information is of greater concern than omission of minor technical details in patient-facing content, underscoring the potential clinical consequences of incomplete online information [28, 29].

Differences observed between YouTube™ and TikTok™ should not be interpreted as indicators of platform superiority. Short-form platforms such as TikTok™ impose inherent structural constraints on content length, and commonly used quality assessment tools, including DISCERN and JAMA, may inherently favor longer and more comprehensive videos [30, 31]. Accordingly, lower scores observed in short-form videos may partially reflect format limitations rather than inaccurate content alone. Moreover, platform-specific algorithms prioritize content based on engagement signals, entertainment value, and regional user behavior rather than educational merit, further complicating cross-platform comparisons [32, 33].

Methodologically, the findings of the present study are consistent with previous evaluations using DISCERN, GQS, JAMA, and VIQI across multiple dental disciplines [34–37]. Across studies, these instruments have repeatedly identified deficiencies in transparency, citation practices, and completeness of online educational content, particularly in videos produced outside academic settings [38–40]. Therefore, the observed associations between engagement metrics and quality scores should be interpreted as correlational rather than causal, reflecting algorithmic prioritization and platform architecture rather than deliberate misinformation or educational intent.

Several limitations should be acknowledged. First, the relatively small sample size (*n* = 38) may limit statistical power and restrict generalizability across languages, regions, and uploader categories. Second, the analysis was limited to English- and Turkish-language content, and videos in other languages may present different quality profiles. Third, due to platform-specific constraints, engagement metrics could not be fully standardized across YouTube™ and TikTok™, precluding direct quantitative comparison of all engagement indicators. In particular, video duration data were not consistently accessible for TikTok™ videos, preventing duration-adjusted analyses and evaluation of potential duration-related bias. Although video duration data were available for YouTube™, duration-adjusted analyses were not performed to avoid overinterpretation and maintain consistency with the study’s cross-platform descriptive design. Fourth, the search strategy relied on technical terminology related to subperiosteal implants, which may have preferentially captured professionally oriented content while underrepresenting patient-generated videos using more colloquial language. Fifth, searches were conducted from Turkey using a local IP address, and retrieved content may therefore reflect region-specific algorithmic behavior, particularly on TikTok™. Finally, although validated assessment tools were used and inter-rater reliability was high, all quality scoring systems inherently involve some degree of subjectivity.

Despite these limitations, this study provides a timely and clinically relevant contribution by highlighting the disconnect between visibility and educational value in social media content related to advanced implant procedures. The findings underscore the urgent need for high-quality, patient-centered, and algorithm-aware educational resources to ensure that online information supports informed decision-making rather than reinforcing misinformation or unrealistic expectations.

## Conclusion

This study provides a descriptive evaluation of the educational quality and reliability of YouTube™ and TikTok™ videos related to subperiosteal implant procedures. As a cross-sectional observational analysis, the findings describe patterns in available online content rather than direct effects on patient behavior, misinformation, or clinical decision-making. The results indicate variable and often limited informational completeness across platforms and uploader categories, while engagement metrics were not associated with measures of informational reliability. Future studies incorporating user-centered outcomes and longitudinal designs are warranted to further explore the implications of online implant-related information.

## Data Availability

All data generated and analyzed in this study are included within this published article and its supplementary files. Additional datasets are available from the corresponding author upon reasonable request.

## Abbreviations

CAD/CAM: Computer-aided design / Computer-aided manufacturing
CI: Confidence interval
DISCERN: Quality assessment instrument for consumer health information
GQS: Global quality scale
ICC: Intraclass correlation coefficient
JAMA: Journal of the American medical association benchmark criteria
Q1–Q3: Interquartile range (First to third quartile)
SD: Standard deviation
VIQI: Video information and quality index
